# Investigation of Tuberculosis in a High School — San Antonio, Texas, 2012

**DOI:** 10.15585/mmwr.mm6431a5

**Published:** 2015-08-14

**Authors:** Tommy L. Camden, Dora Maruffo, Norma Santos, John J. Nava, Carlos Alcantara

**Affiliations:** 1 San Antonio Metropolitan Health District, San Antonio, Texas; 2 Division of Tuberculosis Elimination, National Center for HIV/AIDS, Viral Hepatitis, STD, and TB Prevention, CDC

On February 21, 2012, the San Antonio Metropolitan Health District (SAMHD) Tuberculosis Clinic was notified that two students at Madison High School had laboratory-confirmed pulmonary tuberculosis (TB). During March–September 2012, public health officials from SAMHD collaborated with the school district to conduct an outbreak investigation that included performing tuberculin skin tests (TSTs) on high-risk contacts of active TB patients. To ensure compliance, all TSTs were performed at the school. Initial screening was conducted as soon as a contact was identified and was followed by a second TST ≥8 weeks after the patients with active TB were removed from the school. All positive TSTs were confirmed with an interferon gamma release assay (IGRA) (T-Spot.TB, Oxford Immunotec, Inc.) performed by SAMHD laboratory services (1). IGRA tests can provide additional evidence of infection to encourage acceptance and adherence of foreign-born patients who believe their positive TST is attributable to Bacille Calmette-Guerin vaccination and might also prompt greater acceptance of treatment for latent TB infection compared with a positive TST alone.

Overall, 400 students and 26 faculty members received TSTs. As a result of screening, a third student with active pulmonary TB was identified on April 3, and nine cases of latent TB infection were diagnosed. Because most of these students were initially tested as the school year was ending, follow-up testing for most of them was completed by June 7, after school was officially closed for the summer. However, those students who did not have follow-up testing by that date were tested at the beginning of the school year in September 2012. After identification of the third case, the contact investigation was extended beyond the school to include family members and close friends of all patients. No additional cases of TB or latent TB infection were identified.

All three patients with active TB were symptomatic and had abnormal chest radiographs. All were smear-positive and had positive nucleic acid amplification tests, and the diagnosis of TB was confirmed by culture and IGRA. Active TB patients were started on treatment with two or more anti-TB medications (2–4), and laboratory isolates were sent to the California Department of Public Health Microbial Diseases Laboratory Branch, which is contracted by CDC to perform genotyping. Two of the patients had a history of travel to Vietnam several years earlier; isolates from those two patients were genotypically linked and part of a cluster with similar genotype patterns in other parts of the United States. The isolate from the third patient, who had a history of travel to Africa, was one of only two genotypically identical isolates in the United States.

Interrupting the chain of disease transmission is a critical function of local health departments. The outbreak in this high school led to widespread media attention and concern among students, parents, and the community. In addition to SAMHD evaluating possibly exposed persons and assisting patients to complete the prolonged treatment course, partnering between school and public health officials was crucial to the investigation and management of this outbreak. This partnership ensured communication with the public as the investigation progressed and was facilitated through television and radio interviews, health advisories, press releases, factsheets, and timely bilingual English-Spanish updates developed for the faculty, students, and parents. Challenges included ensuring that the contact investigation and screening activities continued during the summer break and following up on students who had graduated. Developing these partnerships at the local level is important for implementing rapid and effective public health responses to community or school outbreaks.
